# Facile Preparation of a Transparent, Self-Healing, and Recyclable Polysiloxane Elastomer Based on a Dynamic Imine and Boroxine Bond

**DOI:** 10.3390/polym16091262

**Published:** 2024-05-01

**Authors:** Peng Wang, Zhuochao Wang, Wenxin Cao, Jiaqi Zhu

**Affiliations:** 1 College of Mechanics and Engineering Science, Hohai University, Nanjing 211100, China; 2 National Key Laboratory of Science and Technology on Advanced Composites in Special Environments, Harbin Institute of Technology, Harbin 150080, China; 20s118208@stu.hit.edu.cn (Z.W.); zhujq@hit.edu.cn (J.Z.)

**Keywords:** self-healing, polysiloxane, elastomers, imine bonds, boroxine

## Abstract

Transparent polysiloxane elastomers with good self-healing and reprocessing abilities have attracted significant attention in the field of artificial skin and flexible displays. Herein, we propose a simple one-pot method to fabricate a room temperature self-healable polysiloxane elastomer (HPDMS) by introducing dynamic and reversible imine bonds and boroxine into polydimethylsiloxane (PDMS) networks. The presence of imine bonds and boroxine is proved by FT−IR and NMR spectra. The obtained HPDMS elastomer is highly transparent with a transmittance of up to 80%. The TGA results demonstrated that the HPDMS elastomer has good heat resistance and can be used in a wide temperature range. A lower glass transition temperature (*T_g_*, −127.4 °C) was obtained and revealed that the elastomer is highly flexible at room temperature. Because of the reformation of dynamic reversible imine bonds and boroxine, the HPDMS elastomers exhibited excellent autonomous self-healing properties. After healing for 3 h, the self-healing efficiency of HPDMS reached 96.3% at room temperature. Moreover, the elastomers can be repeatedly reprocessed multiple times under milder conditions. This work provides a simple but effective method to prepare transparent self-healable and reprocessable polysiloxane elastomers.

## 1. Introduction

Self-healing materials represent a novel class of intelligent materials. They are endowed with the remarkable ability to autonomously repair damage and restore functionality. Consequently, they offer numerous advantages, including prolonged service life, reduced maintenance costs, and minimized waste generation and pollution levels, as well as enhanced usage safety. Over the last decades, self-healing materials have gained much attention from researchers in various fields, such as engineering, electronics, energy, and the environment [1]. Broadly speaking, self-healing materials can be categorized into two types: extrinsic and intrinsic. The extrinsic type involves embedding microcapsules or microvascular tubes containing healing agents within the material matrix. However, the self-healing efficiency in this case is contingent upon the type and mobility of the healing agent, and the healing time is often limited. On the other hand, intrinsic self-healing materials leverage reversible physical and chemical interactions within the material system. This approach relies solely on polymer diffusion and internal reversible interactions, eliminating the need for externally added healing agents [2]. Remarkably, the healing process in intrinsic materials can be repeated numerous times. Consequently, intrinsic self-healing materials have garnered increasing attention in recent years [3].

A polysiloxane elastomer, an essential organosilicon material, is renowned for its distinct physicochemical properties that encompass high elasticity, robust chemical stability, nontoxicity, and biocompatibility. These attributes have led to its widespread application in diverse fields, including flexible sensors, electronic skins, and biomedical uses [4]. Nevertheless, traditional polysiloxane elastomers often suffer from limited mechanical performance, rendering them vulnerable to damage during operation. Additionally, the inherent irreversible crosslinking structure within these materials precludes the restoration of their original functionality once damaged. Consequently, the development of self-healable, recyclable, and reprocessable polysiloxane elastomers is of utmost importance. This pursuit holds significant potential in extending their service life, minimizing resource wastage, and contributing to a more sustainable future [5,6].

In recent years, extensive efforts have been made to design an intrinsic self-healing polysiloxane elastomer by introducing dynamic non-covalent interaction (such as hydrogen bonds [7,8], ionic interactions [9], π−π stacking [10], host−guest interactions [11], and metal−ligand interactions [12,13]) or reversible covalent bonds (such as imine bonds [14,15], Diels−Alder reactions [16,17], and disulfide exchange [18,19]) into polysiloxane networks. Among them, the imine bond is a typical dynamic bond in organic chemistry, which can be obtained by the reaction of the aldehyde group and amino group under mild conditions. This bond can endow a polysiloxane elastomer with self-healing ability and recyclability [20]. For instance, Yu et al. [21] and Wang et al. [22] reported a transparent, stretchable, and self-healing PDMS elastomer based on imine bonds, respectively. Similarly, in our previous work, we obtained a self-healing PDMS elastomer by mixing amine-functionalized PDMS and 1,4-diformylbenzene (DFB) at room temperature [23]. These elastomers exhibited impressive light transmittance, high stretchability, and remarkable self-healing capabilities. However, their mechanical properties are relatively poor due to the incorporation of dynamic imine bonds. It is necessary to develop a PDMS elastomer with good self-healing performance and desired mechanical strength. Boroxine is formed through the dehydration between three boronic acid molecules and possesses high bond dissociation energy. Due to its unique tripodal ring structure, it can provide a higher crosslink density to improve the mechanical properties of the materials [24]. Additionally, the formation of the boroxine structure is dynamically reversible, making it suitable for the preparation of high-strength self-healing and recyclable PDMS elastomers. Researchers have carried out extensive explorations in the development of self-healing materials based on the boron oxygen hexagonal structure, which often exhibits excellent mechanical performance [25,26]. Recently, many self-healing PDMS elastomers based on dynamic–covalent boroxine bonds have been reported [27,28,29,30]. In our previous studies, a self-healable and reprocessable polysiloxane elastomer was prepared by introducing exchangeable imine bond and boroxine into PDMS networks [31]. However, the precursors are generally not easily available, and the fabrication procedures are time consuming and inefficient. Thus, it is still a challenge to develop an efficient strategy for the preparation of polysiloxane elastomers with good mechanical properties and excellent self-healing.

Herein, a transparent self-healable and recyclable PDMS elastomer was prepared by incorporating a reversible imine bond and boroxine into PDMS networks using a simple and efficient one-pot strategy. This method can minimize chemical waste, save time, and simplify the actual operation. It is of great significance to resource conservation and environmental protection. The presence of an imine bond and boroxine was proved by FT−IR and NMR results. The mechanical and self-healing properties of the elastomer were tested. The key raw material used in this study is an amine-functionalized polydimethylsiloxane (PDMS−NH_2_), which is one of the common commercial softening finishing agents. Compared with amino-terminated PDMS, the material used in this paper is cheaper, easier to obtain, and more suitable for large-scale production and application. The as-prepared PDMS elastomer showed good light transmittance, desirable mechanical properties, and excellent self-healing properties. This research provides new insights into designing self-healing, mechanically tough, and transparent PDMS elastomers. We anticipate that the self-healing elastomer could have potential applications in intelligent sensors and biomedicine (tissue adhesives, agents for drug or cell delivery).

## 2. Materials and Methods

### 2.1. Materials

Amino-modified polydimethylsiloxane (XIAMETER OFX−8040 fluid, viscosity: 800−5000 cPs, nitrogen content: 0.32−0.42%) was obtained from Dow Corning Corporation (Midland, MI, USA). 4-formylphenylboric acid (FPBA) was purchased from Aladdin Chemical Ltd. (Ontario, CA, USA) Methanol was provided by Sigma−Aldrich (St. Louis, MO, USA). All solvents and chemicals were used as received without further purification.

### 2.2. Preparation of the Self-Healing PDMS Elastomer

The PDMS elastomer was prepared via a one-pot method. Firstly, different amounts of 4-formylphenylboric acid (FPBA) were dissolved in 4 mL of methanol and stirred for 2 h. Subsequently, 4 g of amino-modified PDMS (PDMS−NH_2_) was added to the above solution and stirred at room temperature. After several minutes, the polymer solution turned viscous, and crosslinked organogels were obtained. The resulting product was then placed in a fume hood for 12 h to evaporate the solvent, followed by drying in a vacuum oven at 60 °C for 6 h to remove the residual solvent. The product was collected into a polytetrafluoroethylene (PTFE) mold. Finally, the self-healable PDMS elastomers were obtained by mold pressing at room temperature.

### 2.3. Characterization

Fourier-transform infrared spectra (FT−IR) were recorded on a PerkinElmer Frontier (PerkinElmer, Waltham, MA, US) from 4000 to 400 cm^−1^ with 32 scans at a resolution of 4 cm^−1^. Proton nuclear magnetic resonance spectroscopy (^1^H NMR) was obtained using a Bruker 300 MHz spectrometer (Bruker, Bremen, Germany) with CDCl_3_ as the solvent. The transmittance of elastomers was characterized on a UV−Vis spectrophotometer (TU−1810, Beijing Puxi Instrument Factory, Beijing, China). Thermal gravimetric analysis (TGA) was performed on a synchronous thermal analyzer (STA449F3, Netzsch, Selb, Germany) by heating the samples from room temperature to 800 °C at a heating rate of 10 °C/min under the protection of a nitrogen atmosphere. Differential scanning calorimetry (DSC) measurements were conducted on a NETZSCH STA449F3 instrument (Netzsch, Selb, Germany) at a heating rate of 10 °C/min from −140 to 100 °C under a nitrogen atmosphere. Rectangular specimens (40 mm × 10 mm × 2 mm) were formed and used to test the mechanical properties of PDMS elastomers on a universal electronic tension testing machine (Instron 5944, Instron, Norwood, MA, USA) with a strain rate of 20 mm/min at room temperature. The self-healing process of the crack on the elastomer was observed using optical microscopy (WMP−6880, Wumo Optical, Shanghai, China).

### 2.4. Evaluation of Self-Healing Properties

The original PDMS elastomers were cut into two halves using blades. Then, the separated samples were put together under ambient conditions without exterior pressure and heating. After self-healing at room temperature for different times, the tensile strength and elongation of elastomers were obtained from the tensile test. The self-healing efficiency (*HE*) was calculated in Equation (1) as follows:

(1) $$HE=\frac{\sigma_{h}}{\sigma_{0}}\times 100\%$$

where $\sigma_{h}$ and $\sigma_{0}$ correspond to the tensile strength for the healed and original elastomer, respectively.

## 3. Results and Discussion

### 3.1. Preparation and Characterization of the HPDMS Elastomer

**Figure 1:**
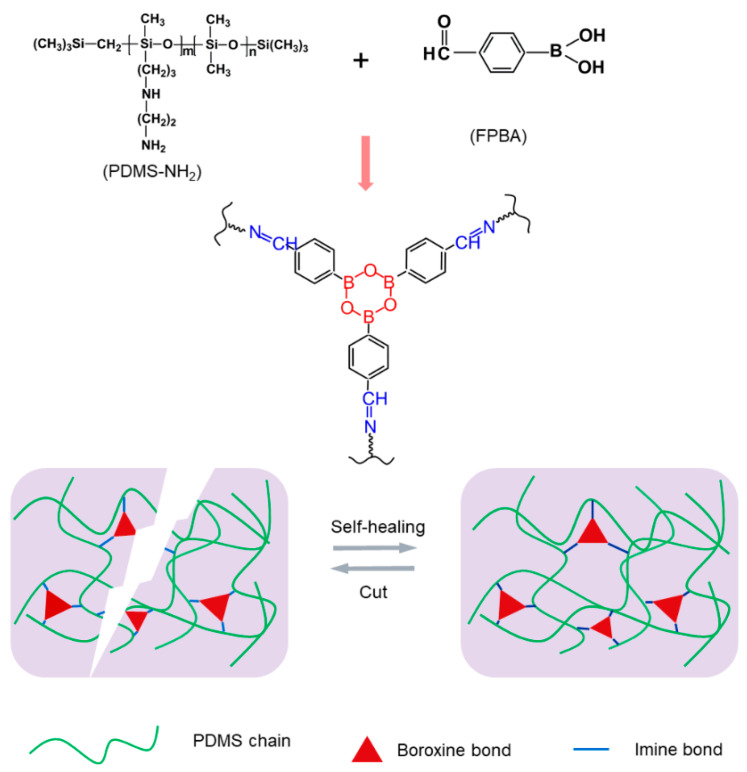
Schematic illustration of the HPDMS elastomer synthesis and the self-healing process.

The chemical structure and the synthesis process of the HPDMS elastomer are presented in Figure 1. The self-healing PDMS elastomer was prepared with PDMS−NH_2_ as a soft segment and FPBA as crosslinker by introducing two dynamic bonds, weak imine bonds, and a strong boroxine structure into the polymer networks. Imine bonds were constructed through a Schiff base reaction between the amine groups in the PDMS backbone and the aldehyde groups of FPBA. Meanwhile, boroxine was formed through a dehydration reaction between the boron hydroxyl groups of FPBA. Thus, the crosslinking density of this material can be easily tunable through the ratio of PDMS−NH_2_ and FPBA. The dynamic and reversible imine bonds and boroxine can not only provide crosslinking points for improving the tensile strength and stretchability of the material but also endow the elastomer with fast autonomous self-healing ability at room temperature.

**Figure 2:**
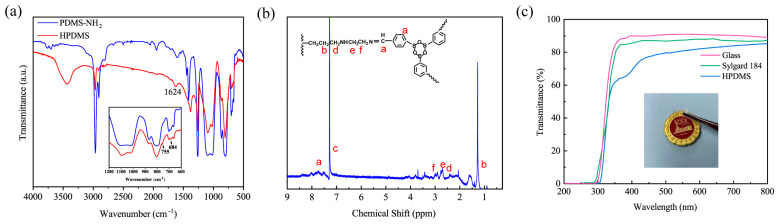
(**a**) FT−IR spectra of PDMS−NH_2_ and the HPDMS elastomer. (**b**) ^1^H NMR spectra of the HPDMS elastomer. (**c**) UV−Vis transmittance spectra of the glass slide, Sylgard 184, and HPDMS elastomer.

The successful synthesis of the HPDMS elastomer was evidenced by the FT−IR and ^1^H NMR spectra. As depicted in Figure 2a, the spectrum of the HPDMS elastomer shows similar peaks as PDMS−NH_2_. The peak at 800 cm^−1^ is attributed to the stretching vibration of Si−C. The doublet at 1019 cm^−1^ and 1096 cm^−1^ is assigned to the asymmetric stretching of Si−O-Si in the backbone. The adsorption peak at 2965 cm^−1^ is due to the C−H stretching vibration from the alkyl group. Compared with PDMS−NH_2_, in the spectrum of HPDMS, a peak corresponding to the C=N stretching vibration appeared at 1624 cm^−1^, and two characteristic absorption peaks of the boroxine structure emerged at 746 and 687 cm^−1^. This observation suggests the successful formation of imine bonds and boroxine structures [29]. The ^1^H NMR spectrum of HPDMS is provided in Figure 2b. The chemical shift peaks at b (δ = 1.27 ppm), d, e, and f (δ = 2.62–2.93 ppm) correspond to the methyl (-Si−CH_3_) and methylene (−Si−CH_2_CH_2_CH_2_−) groups attached to silicon. Additionally, signals at c (δ = 7.3 ppm) and a (δ = 7.98 ppm) belong to the protons from the benzene ring and imine bond (−HC=N−), respectively [27]. These findings indicate the successful construction of imine bonds and boroxine structures through the Schiff base reaction and dehydration reaction between PDMS−NH_2_ and FPBA. The above results prove the successful preparation of self-healing PDMS by introducing imine bonds and boroxine into the PDMS networks.

The optical transparency of self-healing materials greatly determines their application in flexible displays, solar cells, artificial skin, medical supplies, and others. Currently, most healable PDMS elastomers are chromatic and opaque due to the presence of metal ions and chromophores. The transparency of HPDMS elastomers is evaluated using a UV−Vis spectrophotometer. As shown in Figure 2c, the self-healing PDMS elastomer exhibits good transparency. The average transmittance of the HPDMS elastomer with a thickness of 1 mm is 80% in the visible light region (400–800 nm), and the underneath images can be clearly seen through the elastomer, although the transparency was lower than that of quartz glass (≈91%). The value was comparable to the transparency of commercial Sylgard 184 (≈87%) and other transparent elastomers [32,33].

**Figure 3:**
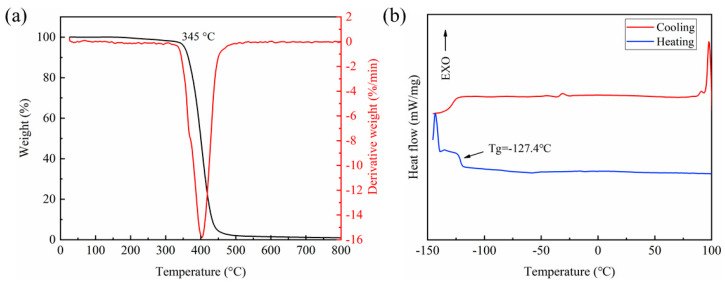
(**a**) TGA curves and (**b**) DSC traces of the HPDMS elastomer.

Thermogravimetric analysis (TGA) was performed in a nitrogen atmosphere to determine the thermal stability and decomposition temperature of the elastomers. It can be seen from the TGA curves in Figure 3a that there is only one weightlessness from 300 to 600 °C. This significant weight loss mainly corresponds to the depolymerization of the siloxane chains [34]. This stage indicates that the HPDMS elastomer is converted into cyclic oligomeric species due to the thermally activated depolymerization mechanism [35]. The decomposition temperature (the temperature at 5% weight loss) of the self-healing PDMS elastomer is 345 °C, which is higher than that of the PDMS elastomer based on only imine bonds in our previous work [23]. This is due to the higher crosslinking density and introduction of boroxine with high bond dissociation energy. These results indicate that the self-healing elastomers have good heat resistance and thermal stability and can be used in a wide temperature range.

In order to investigate the thermally amendable characteristic of the HPDMS elastomer, a differential scanning calorimetry (DSC) analysis was conducted via a heating and cooling cycle in the temperature range of −150–100 °C (Figure 3b). It can be observed that there are no endothermic or exothermic peaks during heating and cooling cycles (the peaks at −143 °C and 100 °C are not genuine, and they may be due to an experimental error), which indicates that the HPDMS elastomer does not have a melting temperature (*T_m_*) and crystallization temperature (*T_c_*). The polymer is amorphous. Additionally, an inflection point in the heating process was observed and taken as the glass transition temperature (*T_g_*). The *T_g_* of HPDMS is about −127.4 °C, which indicates that the elastomer is highly flexible at room temperature [21]. Such low *T_g_* benefits the movement of polymer chains, promoting chain diffusion, bond exchange, and the re-entanglement of molecular chains at the fracture interface [36]. It is helpful for self-healing at room temperature.

### 3.2. Mechanical Properties of the HPDMS Elastomer

**Figure 4:**
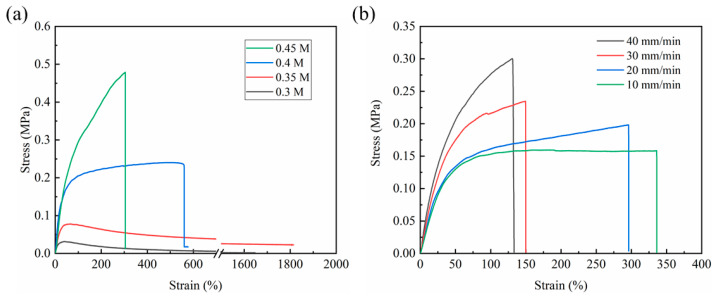
Stress−strain curves of the HPDMS elastomer (**a**) with different FPBA concentrations and (**b**) different stretching speeds.

The mechanical properties of the HPDMS elastomer with different FPBA contents were investigated by tensile test at room temperature. It can be seen in Figure 4a that HPDMS exhibited different tensile strengths and elongations with varying FPBA concentrations. When the FPBA concentration was 0.3 M, the polymer behaved like a viscous liquid and could not form an elastic elastomer due to insufficient crosslinking sites. Therefore, although it had a higher elongation (>1800%), the tensile strength was too low (<2 kPa). With the increase in FPBA concentration, the mechanical strength markedly improved. When the FPBA concentration was 0.4 M, a three-dimensional polymer network was constructed, resulting in tensile strength and elongation at a break of 0.24 MPa and 554%, respectively. However, when the FPBA concentration was further increased to 0.45 M, although the tensile strength increased to 0.47 MPa, the elongation reduced to 304%. This is because a higher FPBA concentration leads to the formation of more imine bonds and boroxine, providing a higher crosslinking degree, which is beneficial for improving the tensile strength of the polymer. However, highly crosslinked polymer networks also limit chain migration and segment dynamics, leaving insufficient space in the polymer network for polymer chain slippage under stretching, resulting in quick fracture of the networks and showing a lower elongation at the break [37]. The mechanical properties can be regulated by adjusting the concentration of FPBA. Moreover, the mechanical strength is higher than that of PDMS based on the imine bond alone in our previous work [23] due to the introduction of stronger boroxine with higher bond energy in the polymer. This finding is consistent with the results of TGA. Furthermore, the mechanical performances of the HPDMS elastomer under different stretching speeds were also compared. As shown in Figure 4b, the stretchability of HPDMS is related to the stretching speed during tensile tests. With the stretching speed increasing from 10 mm/min to 40 mm/min, the tensile strength increased from 0.15 MPa to 0.30 MPa, while the elongation at the break decreased from 336% to 130%. A higher stretching speed resulted in higher tensile strength but lower elongation at the break. This phenomenon could be ascribed to the fact that when a higher stretching speed is applied, the movement of polymer chains cannot keep up with the external force, allowing less time for the displacement, reorientation, and reconfiguration of polymer chain segments and the reformation of the broken dynamic covalent bond, which reduces the fracture tolerance [38]. Conversely, when the elastomer was applied with a lower stretching speed, there was enough time for the relaxation and configuration of polymer chains, and it can be compensated for the breakage of dynamic covalent bonds through a slip between molecular chains. As a result, the HPDMS elastomer shows higher extensibility at a lower stretching speed.

### 3.3. Self-Healing Properties of the HPDMS Elastomer

**Figure 5:**
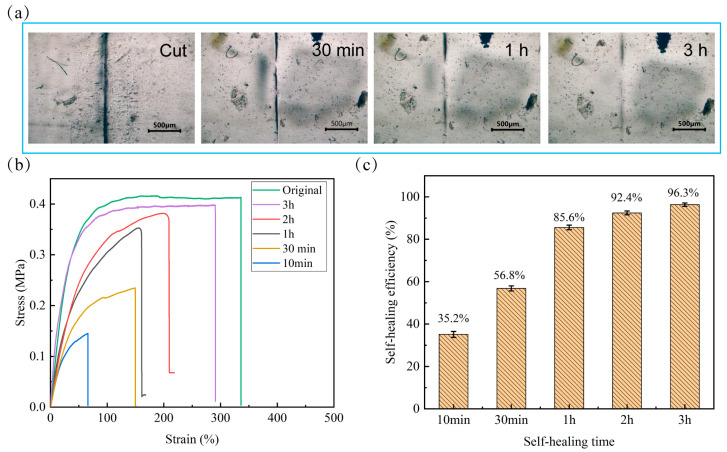
The self-healing properties of the elastomers: (**a**) optical microscope images, (**b**) stress−strain curves, and (**c**) self-healing efficiency of the HPDMS elastomer healed at room temperature for different times.

The self-healing performance of the HPDMS elastomer was observed and evaluated. The self-healing process was monitored through optical microscopic observation. As shown in Figure 5a, a crack was created on the surface of the HPDMS elastomer using a razor blade, and the incision was very apparent before healing. As the healing time increased, the incision gradually became narrower and shallower. After healing at room temperature for 3 h, the incision on the elastomer almost disappeared, demonstrating the excellent self-healing ability of HPDMS. In addition, the tensile experiments were conducted to quantitatively evaluate the self-healing properties of HPDMS. First, HPDMS strips were cut into two pieces. Then, the contacting surfaces of the two pieces were placed together for self-healing at room temperature without any external stimuli. Figure 5b shows the stress−strain curves of the HPDMS elastomer healed at room temperature at different times. It can be seen that the original samples exhibited a tensile strength and an elongation at a break of 0.41 MPa and 335%, respectively. The mechanical properties of healed elastomer increased with the increase in healing time. After healing for 30 min, the tensile strength and elongation can recover to 56% and 44%, respectively. When the severed samples were healed for 3 h, the tensile strength and elongation at the break of HPDMS can reach 0.39 MPa and 335%, respectively. These values are very close to the original samples. Furthermore, self-healing efficiencies (*HE*) calculated from the ratio of the tensile strength of the healed and original samples were also used to evaluate the self-healing performance. It can be seen in Figure 5c that the *HE* is related to the self-healing time. When the self-healing time increased from 10 min to 3 h, the average *HE* of HPDMS increased from 35.2% to 96.3%. Although the *HE* of HPDMS is slightly lower than the elastomer (whose *HE* is 99% when healing for 1 h) we reported previously [23], it is still higher than many other PDMS elastomers [7,39,40,41]. All these results indicate that the prepared HPDMS elastomer exhibits good autonomous self-healing capability. This was the result of the spontaneous and rapid reconstruction of dynamic imine bonds and reversible boroxine at room temperature. Additionally, the flexibility of the molecular chains of HPDMS was also beneficial for the rapid integration of the fracture surfaces.

### 3.4. Reprocessability of the HPDMS Elastomer

**Figure 6:**
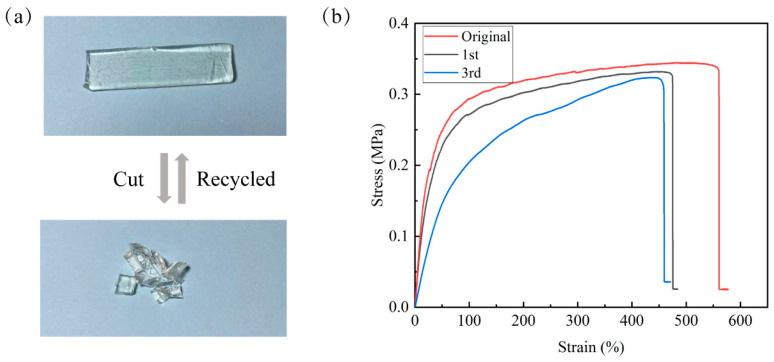
(**a**) Photographs of the samples were cut into small pieces and reprocessed by pressing at room temperature. (**b**) The stress−strain curves of the original and regenerated HPDMS elastomer.

In general, a conventional PDMS elastomer cannot be recycled and reprocessed due to the presence of permanent covalent crosslinked network structures within the polymer, leading to resource waste and environmental pollution [42]. Introducing dynamic reversible chemical bonds into PDMS can endow materials with recycling and reprocessing ability. The reprocessability of the HPDMS elastomer was evaluated through the following procedures. Firstly, the rectangular sample was cut into small pieces, which were then pressed in a Teflon mold at room temperature at a pressure of ~10 MPa for several hours, to reshape it (Figure 6a). The stress−strain curves of original and recovered samples are shown in Figure 6b. The original samples exhibited a tensile strength of 0.34 MPa and an elongation at a break of 560%. Although the elongation slightly decreased with an increase in the number of cycles, the mechanical strength could almost reach the value of the original elastomer. Even after three cycles of cutting/recycling process, the tensile strength and elongation of HPDMS still reached 0.32 MPa and 458%, respectively. The recovery ratio of mechanical properties (defined as the ratio of the mechanical property after and before reprocessing) exceeded 94%. These results indicate an excellent reprocessability of HPDMS elastomers under mild conditions. The remarkable reprocessability of HPDMS can be attributed to the reformation of dynamic imine bonds and boroxine in polymers. When the polymer specimen is shredded and then compressed together, Schiff base reactions and the reformation of boroxine could occur between the particle surfaces, reconnecting the grains. As a result, the elastomer can regain its mechanical strength after reprocessing. This manner offers a facile way to reprocess polymers under a milder condition instead of hot pressing, aligning with the trend of environmental protection and resource conservation.

## 4. Conclusions

In summary, a transparent polysiloxane elastomer with good room temperature self-healing and reprocessing abilities was successfully prepared by introducing dynamic imine bonds and boroxine into polymer networks. The elastomer exhibits high optical transparency (80%) in the visible region. The results of DSC and TGA show that the HPDMS elastomer has a low glass transition temperature (−127.4 °C) and a high decomposition temperature (345 °C), indicating that the elastomer is highly flexible at room temperature and possesses good heat resistance and thermal stability. Owing to the dynamic nature of imine bonds and boroxine, the HPDMS elastomer exhibits excellent autonomous self-healing properties. A damaged sample can rapidly recover its mechanical strength after healing for 3 h at room temperature without any external stimuli, such as light or heat. Additionally, the elastomer can be repeatedly reprocessed multiple times under milder conditions without significant degradation in mechanical performance. This work provides a simple and efficient method to prepare a transparent self-healable and recyclable polysiloxane elastomer. It has great potential in the field of flexible electronics, wearable devices, and biomedicine.

## Data Availability

Data are contained within the article.

## References

[1] Wen N., Song T., Ji Z., Jiang D., Wu Z., Wang Y., Guo Z. (2021). Recent advancements in self-healing materials: Mechanicals, performances and features. React. Funct. Polym..

[2] Li B., Cao P., Saito T., Sokolov A.P. (2023). Intrinsically Self-Healing Polymers: From Mechanistic Insight to Current Challenges. Chem. Rev..

[3] Utrera-Barrios S., Verdejo R., López-Manchado M.A., Hernández Santana M. (2020). Evolution of self-healing elastomers, from extrinsic to combined intrinsic mechanisms: A review. Mater. Horiz..

[4] Ariati R., Sales F., Souza A., Lima R.A., Ribeiro J. (2021). Polydimethylsiloxane Composites Characterization and Its Applications: A Review. Polymers.

[5] Ye J., Jin H., Zu Z., Yu B., Xiang H., Zhang M. (2023). Dynamic Crosslinked Silicones and their Composites: A Review. Eng. Sci..

[6] Kowalewska A., Majewska-Smolarek K. (2024). Synergistic Self-Healing Enhancement in Multifunctional Silicone Elastomers and Their Application in Smart Materials. Polymers.

[7] Ma W., Yang X., He Y., Lai J., Wang Z., Xie M., Lu X., Xia H. (2023). A self-healing polydimethylsiloxane elastomer with high strength and high modulus. Polymer.

[8] Ciubotaru B.I., Dascalu M., Zaltariov M.F., Macsim A.M., Damoc M., Bele A., Tugui C., Varganici C.D., Cazacu M. (2022). Catalyst-free crosslinked sustainable functional silicones by supramolecular interactions. React. Funct. Polym..

[9] Zheng S., Chen Y., Brook M.A. (2020). Thermoplastic silicone elastomers based on Gemini ionic crosslinks. Polym. Chem..

[10] Mei J., Jia X., Lai J., Sun Y., Li C., Wu J., Cao Y., You X., Bao Z. (2016). A Highly Stretchable and Autonomous Self-Healing Polymer Based on Combination of Pt···Pt and π-π Interactions. Macromol. Rapid Commun..

[11] Yoshida D., Park J., Ikura R., Yamashita N., Yamaguchi H., Takashima Y. (2022). Self-healable Poly(dimethyl siloxane) Elastomers Based on Host-guest Complexation between Methylated β-Cyclodextrin and Adamantane. Chem. Lett..

[12] Zhao Z., Zhu S., Li B., Niu C., Tong Y., Ma N., Dong X., Han B., Huang H., Qi M. (2022). A Stiffness Tunable Self-Healing Composite Comprising PDMS and Titanium Dioxide. ACS Appl. Polym. Mater..

[13] Zhang T., Li C., Li W., Wang Z., Gu Z., Li J., Yuan J., Ou-Yang J., Yang X., Zhu B. (2024). A Self-Healing Optoacoustic Patch with High Damage Threshold and Conversion Efficiency for Biomedical Applications. Nano-Micro Lett..

[14] Lei X., Huang Y., Liang S., Zhao X., Liu L. (2020). Preparation of highly transparent, room-temperature self-healing and recyclable silicon elastomers based on dynamic imine bond and their ion responsive properties. Mater. Lett..

[15] Lin Z., Deng H., Hou Y., Liu X., Xu R., Xiang H., Peng Z., Rong M., Zhang M. (2022). Dual-crosslinking side chains with an asymmetric chain structure: A facile pathway to a robust, self-healable, and re-dissolvable polysiloxane elastomer for recyclable flexible devices. J. Mater. Chem. A.

[16] Yilmaz D., Lansade D., Lewandowski S., Perraud S., Llevot A., Carlotti S. (2022). Combination of permanent hydrosilylation and reversible Diels–Alder reactions for self-healing poly(dimethylsiloxane) materials with enhanced ageing properties. Mater. Today Chem..

[17] Zhao L., Shi X., Yin Y., Jiang B., Huang Y. (2020). A self-healing silicone/BN composite with efficient healing property and improved thermal conductivities. Compos. Sci. Technol..

[18] Zhao L., Yin Y., Jiang B., Guo Z., Qu C., Huang Y. (2020). Fast room-temperature self-healing siloxane elastomer for healable stretchable electronics. J. Colloid Interface Sci..

[19] Wang Z., Liu Y., Zhang D., Gao C., Wu Y. (2021). Mussel-inspired self-healing PDMS/AgNPs conductive elastomer with tunable mechanical properties and efficient antibacterial performances for wearable sensor. Compos. Part B.

[20] Yang Z., Li H., Zhang L., Lai X., Zeng X. (2020). Highly stretchable, transparent and room-temperature self-healable polydimethylsiloxane elastomer for bending sensor. J. Colloid Interface Sci..

[21] Zhang B., Zhang P., Zhang H., Yan C., Zheng Z., Wu B., Yu Y. (2017). A Transparent, Highly Stretchable, Autonomous Self-Healing Poly(dimethyl siloxane) Elastomer. Macromol. Rapid Commun..

[22] Sun J., Pu X., Liu M., Yu A., Du C., Zhai J., Hu W., Wang Z. (2018). Self-Healable, Stretchable, Transparent Triboelectric Nanogenerators as Soft Power Sources. ACS Nano.

[23] Wang P., Yang L., Dai B., Yang Z., Guo S., Gao G., Xu L., Sun M., Yao K., Zhu J. (2020). A self-healing transparent polydimethylsiloxane elastomer based on imine bonds. Eur. Polym. J..

[24] Li X., Zhang Y., Shi Z., Wang D., Yang H., Zhang Y., Qin H., Lu W., Chen J., Li Y. (2024). Water-stable boroxine structure with dynamic covalent bonds. Nat. Commun..

[25] Delpierre S., Willocq B., Manini G., Lemaur V., Goole J., Gerbaux P., Cornil J., Dubois P., Raquez J.-M. (2019). Simple Approach for a Self-Healable and Stiff Polymer Network from Iminoboronate-Based Boroxine Chemistry. Chem. Mater..

[26] Bao C., Jiang Y., Zhang H., Lu X., Sun J. (2018). Room-Temperature Self-Healing and Recyclable Tough Polymer Composites Using Nitrogen-Coordinated Boroxines. Adv. Funct. Mater..

[27] Zhang K., Liu Y., Wang Z., Song C., Gao C., Wu Y. (2020). A type of self-healable, dissoluble and stretchable organosilicon elastomer for flexible electronic devices. Eur. Polym. J..

[28] Lai P., Yuan Y., Huang Y., Bai H., Zhou Z., Tang C., Wen J., Liu L. (2023). Preparation of Robust, Room-Temperature Self-Healable and Recyclable Polysiloxanes Based on Hierarchical Hard Domains. Adv. Eng. Mater..

[29] Liang S., Huang Y., Yuan Y., Wang Y., Yang B., Zhao X., Liu L. (2021). Development of a Strong, Recyclable Poly(dimethylsiloxane) Elastomer with Autonomic Self-Healing Capabilities and Fluorescence Response Properties at Room Temperature. Macromol. Mater. Eng..

[30] Wang J., Lai J., Jia X. (2022). Highly stretchable and stretch-induced fluorescence chromism self-healing materials based on boroxine and dynamic imine bond. J. Mater. Chem. C.

[31] Wang P., Wang Z., Liu L., Ying G., Cao W., Zhu J. (2023). Self-Healable and Reprocessable Silicon Elastomers Based on Imine-Boroxine Bonds for Flexible Strain Sensor. Molecules.

[32] Wu J., Han S., Yang T., Li Z., Wu Z., Gui X., Tao K., Miao J., Norford L.K., Liu C. (2018). Highly Stretchable and Transparent Thermistor Based on Self-Healing Double Network Hydrogel. ACS Appl. Mater. Interfaces.

[33] Dang C., Wang M., Yu J., Chen Y., Zhou S., Feng X., Liu D., Qi H. (2019). Transparent, Highly Stretchable, Rehealable, Sensing, and Fully Recyclable Ionic Conductors Fabricated by One-Step Polymerization Based on a Small Biological Molecule. Adv. Funct. Mater..

[34] Kabir I.I., Fu Y., De Souza N., Baena J.C., Yuen A.C.Y., Yang W., Mata J., Peng Z., Yeoh G.H. (2020). PDMS/MWCNT nanocomposite films for underwater sound absorption applications. J. Mater. Sci..

[35] Genovese A., Shanks R.A. (2008). Fire performance of poly(dimethyl siloxane) composites evaluated by cone calorimetry. Compos. Part A.

[36] Dzhardimalieva G.I., Yadav B.C., Kudaibergenov S.E., Uflyand I.E. (2020). Basic Approaches to the Design of Intrinsic Self-Healing Polymers for Triboelectric Nanogenerators. Polymers.

[37] Song F., Li Z., Jia P., Zhang M., Bo C., Feng G., Hu L., Zhou Y. (2019). Tunable “soft and stiff”, self-healing, recyclable, thermadapt shape memory biomass polymers based on multiple hydrogen bonds and dynamic imine bonds. J. Mater. Chem. A.

[38] Lv C., Zhao K., Zheng J. (2018). A Highly Stretchable Self-Healing Poly(dimethylsiloxane) Elastomer with Reprocessability and Degradability. Macromol. Rapid Commun..

[39] Wang L., Cai Y., Zhang H., Zou H., Chen Y., Liang M., Heng Z. (2022). Room-temperature self-healing polysiloxane elastomer with reversible cross-linked network. Polymer.

[40] Zhou X., Gong Z., Fan J., Chen Y. (2021). Self-healable, recyclable, mechanically tough transparent polysiloxane elastomers based on dynamic microphase separation for flexible sensor. Polymer.

[41] Lai J., Wang X., Zhao Q., Zhang C., Gong T., He L., Wang Z., Xia H. (2024). 3D Printing Self-Healing and Self-Adhesive Elastomers for Wearable Electronics in Amphibious Environments. ACS Appl. Mater. Interfaces.

[42] Yan X., Bai L., Feng B., Zheng J. (2022). Mechanically strong, thermally stable, and reprocessable poly(dimethylsiloxane) elastomers enabled by dynamic silyl ether linkages. Eur. Polym. J..

